# Supplementary material for: A UMLS-based spell checker for natural language processing in vaccine safety
Source: BMC Med Inform Decis Mak. 2007 Feb 12;7:3. doi: 10.1186/1472-6947-7-3 (PMC1805499; doi:10.1186/1472-6947-7-3)
Supplement: Additional file 13 — RAP application directory. Sets up directory for RAP (RDF application for PHP) source code [file 1472-6947-7-3-S13.gz › rap/doc/phpdoc/elementindex_ontModel.html]

xml version="1.0" encoding="iso-8859-1"?


## [ontModel] element index

### Package indexes

- model
- dataset
- infModel
- resModel
- syntax
- rdql
- utility

All elements
  

a
b
c
d
e
f
g
h
i
l
m
n
o
p
r
s
t
u
v

a

top

addComment
:   OntResource::addComment() in OntResource.php

    Add the given comment to this resource.

addDomain
:   OntProperty::addDomain() in OntProperty.php

    Add a resource representing the domain of this property.

addIsDefinedBy
:   OntResource::addIsDefinedBy() in OntResource.php

    Add a resource that is declared to provide a definition of this resource.

addLabelProperty
:   OntResource::addLabelProperty() in OntResource.php

    Add the given Label to this resource

addProperty
:   OntResource::addProperty() in OntResource.php

    Add a property to this resource.

addRange
:   OntProperty::addRange() in OntProperty.php

    Add a resource representing the range of this property.

addRDFType
:   OntResource::addRDFType() in OntResource.php

    Add the given class as one of the rdf:type's for this resource.

addSeeAlso
:   OntResource::addSeeAlso() in OntResource.php

    Add a resource that is declared to provided additional information about the definition of this resource.

addSubClass
:   OntClass::addSubClass() in OntClass.php

    Add a sub-class of this class.

addSubProperty
:   OntProperty::addSubProperty() in OntProperty.php

    Add a sub-property of this property.

addSuperClass
:   OntClass::addSuperClass() in OntClass.php

    Add a super-class of this class.

addSuperProperty
:   OntProperty::addSuperProperty() in OntProperty.php

    Add a super-property of this property.

ALL\_DIFFERENT
:   OWLVocabulary::ALL\_DIFFERENT() in OWLVocabulary.php

ALL\_VALUES\_FROM
:   OWLVocabulary::ALL\_VALUES\_FROM() in OWLVocabulary.php

ANNOTATION\_PROPERTY
:   OWLVocabulary::ANNOTATION\_PROPERTY() in OWLVocabulary.php

asClass
:   OntResource::asClass() in OntResource.php

    Answer a view of this resource as a class

asIndividual
:   OntResource::asIndividual() in OntResource.php

    Answer a view of this resource as an Individual

asOntProperty
:   OntResource::asOntProperty() in OntResource.php

    Answer a view of this resource as a property

b

top

BACKWARD\_COMPATIBLE\_WITH
:   OWLVocabulary::BACKWARD\_COMPATIBLE\_WITH() in OWLVocabulary.php

c

top

CARDINALITY
:   OWLVocabulary::CARDINALITY() in OWLVocabulary.php

COMMENT
:   OWLVocabulary::COMMENT() in OWLVocabulary.php

    Answer the predicate that denotes comment annotation on an ontology element.

COMMENT
:   RdfsVocabulary::COMMENT() in RdfsVocabulary.php

    Answer the predicate that denotes comment annotation on an ontology element.

COMPLEMENT\_OF
:   OWLVocabulary::COMPLEMENT\_OF() in OWLVocabulary.php

createIndividual
:   OntModel::createIndividual() in OntModel.php

    Answer a resource that represents an Individual node in this model.

createInstance
:   OntClass::createInstance() in OntClass.php

    Answer a resource that represents an instance of this OntClass and Individual node in this model.

createOntClass
:   OntModel::createOntClass() in OntModel.php

    Answer a resource that represents a class description node in this model.

createOntProperty
:   OntModel::createOntProperty() in OntModel.php

    Answer a resource that represents an OntProperty node in this model.

d

top

DATATYPE
:   OWLVocabulary::DATATYPE() in OWLVocabulary.php

DATATYPE\_PROPERTY
:   OWLVocabulary::DATATYPE\_PROPERTY() in OWLVocabulary.php

DATATYPE\_RESTRICTION
:   OWLVocabulary::DATATYPE\_RESTRICTION() in OWLVocabulary.php

DATA\_RANGE
:   OWLVocabulary::DATA\_RANGE() in OWLVocabulary.php

DEPRECATED\_CLASS
:   OWLVocabulary::DEPRECATED\_CLASS() in OWLVocabulary.php

DEPRECATED\_PROPERTY
:   OWLVocabulary::DEPRECATED\_PROPERTY() in OWLVocabulary.php

DIFFERENT\_FROM
:   OWLVocabulary::DIFFERENT\_FROM() in OWLVocabulary.php

DISJOINT\_WITH
:   OWLVocabulary::DISJOINT\_WITH() in OWLVocabulary.php

DISTINCT\_MEMBERS
:   OWLVocabulary::DISTINCT\_MEMBERS() in OWLVocabulary.php

DOMAIN
:   RdfsVocabulary::DOMAIN() in RdfsVocabulary.php

    Answer the predicate that denotes the domain of a property.

DOMAIN
:   OWLVocabulary::DOMAIN() in OWLVocabulary.php

    Answer the predicate that denotes the domain of a property.

e

top

EQUIVALENT\_CLASS
:   OWLVocabulary::EQUIVALENT\_CLASS() in OWLVocabulary.php

EQUIVALENT\_PROPERTY
:   OWLVocabulary::EQUIVALENT\_PROPERTY() in OWLVocabulary.php

f

top

FUNCTIONAL\_PROPERTY
:   OWLVocabulary::FUNCTIONAL\_PROPERTY() in OWLVocabulary.php

g

top

getComment
:   OntResource::getComment() in OntResource.php

    Answer the comment string for this object. If there is more than one such resource, an arbitrary selection is made.

getDomain
:   OntProperty::getDomain() in OntProperty.php

    Answer a OntClass that represents the domain class of this property.

getInstanceRdfType
:   OntResource::getInstanceRdfType() in OntResource.php

    returns the rdf:type, that this distinct resource is instance of.

getIsDefinedBy
:   OntResource::getIsDefinedBy() in OntResource.php

    Answer a resource that is declared to provide a definition of this resource.

getLabelProperty
:   OntResource::getLabelProperty() in OntResource.php

    Answer the label ResLiteral for this object.

getPropertyValue
:   OntResource::getPropertyValue() in OntResource.php

    Answer the value of a given RDF property for this resource as $returnType, or null if it doesn't have one. If there is more than one RDF statement with the given property for the current value, it is not defined which of the values will be returned.

getRange
:   OntProperty::getRange() in OntProperty.php

    Answer a OntClass that represents the range class of this property.

getRDFType
:   OntResource::getRDFType() in OntResource.php

    Answer the rdf:type (ie the class) of this resource.

getSeeAlso
:   OntResource::getSeeAlso() in OntResource.php

    Answer a resource that provides additional information about this resource.

getSubClass
:   OntClass::getSubClass() in OntClass.php

    Answer a class that is the sub-class of this class.

getSubProperty
:   OntProperty::getSubProperty() in OntProperty.php

    Answer a property that is the sub-property of this property.

getSuperClass
:   OntClass::getSuperClass() in OntClass.php

    Answer a class that is the super-class of this class.

getSuperProperty
:   OntProperty::getSuperProperty() in OntProperty.php

    Answer a property that is the super-property of this property.

getVocabulary
:   OntResource::getVocabulary() in OntResource.php

    Answer a reference to the ontology language profile that governs the ontology model to which this ontology resource is attached.

h

top

hasComment
:   OntResource::hasComment() in OntResource.php

    Answer true if this resource has the given comment.

hasDomain
:   OntProperty::hasDomain() in OntProperty.php

    Answer true if the given resource a class specifying the domain of this property.

hasLabelProperty
:   OntResource::hasLabelProperty() in OntResource.php

    Answer true if this resource has the given label.

hasRange
:   OntProperty::hasRange() in OntProperty.php

    Answer true if the given resource a class specifying the range of this property.

hasRDFType
:   OntResource::hasRDFType() in OntResource.php

    Answer true if this resource has the given rdf:type.

hasSeeAlso
:   OntResource::hasSeeAlso() in OntResource.php

    Answer true if this resource has the given resource as a source of additional information.

hasSubclass
:   OntClass::hasSubclass() in OntClass.php

    Answer true if the given class is a sub-class of this class.

hasSubProperty
:   OntProperty::hasSubProperty() in OntProperty.php

    Answer true if the given property is a sub-property of this property.

hasSuperClass
:   OntClass::hasSuperClass() in OntClass.php

    Answer true if the given class is a super-class of this class.

hasSuperProperty
:   OntProperty::hasSuperProperty() in OntProperty.php

    Answer true if the given property is a super-property of this property.

HAS\_VALUE
:   OWLVocabulary::HAS\_VALUE() in OWLVocabulary.php

i

top

IMPORTS
:   OWLVocabulary::IMPORTS() in OWLVocabulary.php

INCOMPATIBLE\_WITH
:   OWLVocabulary::INCOMPATIBLE\_WITH() in OWLVocabulary.php

Individual
:   Individual::Individual() in Individual.php

    Constructor You can supply a uri

Individual
:   Individual in Individual.php

    Interface that encapsulates an individual in an ontology, sometimes referred to as a fact or assertion.

INTERSECTION\_OF
:   OWLVocabulary::INTERSECTION\_OF() in OWLVocabulary.php

INVERSE\_FUNCTIONAL\_PROPERTY
:   OWLVocabulary::INVERSE\_FUNCTIONAL\_PROPERTY() in OWLVocabulary.php

INVERSE\_OF
:   OWLVocabulary::INVERSE\_OF() in OWLVocabulary.php

isDefinedBy
:   OntResource::isDefinedBy() in OntResource.php

    Answer true if this resource is defined by the given resource.

IS\_DEFINED\_BY
:   RdfsVocabulary::IS\_DEFINED\_BY() in RdfsVocabulary.php

    Answer the predicate that denotes isDefinedBy annotation on an ontology element

IS\_DEFINED\_BY
:   OWLVocabulary::IS\_DEFINED\_BY() in OWLVocabulary.php

    Answer the predicate that denotes isDefinedBy annotation on an ontology element

Individual.php
:   Individual.php in Individual.php

l

top

LABEL
:   RdfsVocabulary::LABEL() in RdfsVocabulary.php

    Answer the predicate that denotes label annotation on an ontology element

LABEL
:   OWLVocabulary::LABEL() in OWLVocabulary.php

    Answer the predicate that denotes label annotation on an ontology element

listClasses
:   OntModel::listClasses() in OntModel.php

    Answer an array that ranges over all of the various forms of class description resource in this model.

listComments
:   OntResource::listComments() in OntResource.php

    Answer an array of all of the comment literals for this resource.

listDomain
:   OntProperty::listDomain() in OntProperty.php

    Answer an array of all of the declared domain classes of this property.

listInstances
:   OntClass::listInstances() in OntClass.php

    Answer an ResIterator over the individuals in the model that have this class among their types.

listIsDefinedBy
:   OntResource::listIsDefinedBy() in OntResource.php

    Answer an array of all of the resources that are declared to define this resource.

listLabelProperties
:   OntResource::listLabelProperties() in OntResource.php

    Answer an array of all of the label literals for this resource, with the given language, if $language is set.

listProperty
:   OntResource::listProperty() in OntResource.php

    Answer an array of values of a given RDF property for this resource as $returnType, or null if it doesn't have one.

listRange
:   OntProperty::listRange() in OntProperty.php

    Answer an array of all of the declared range classes of this property.

listRDFTypes
:   OntResource::listRDFTypes() in OntResource.php

    Answer an array of the RDF classes to which this resource belongs.

listSeeAlso
:   OntResource::listSeeAlso() in OntResource.php

    Answer an array of all of the resources that are declared to provide addition information about this resource.

listSubClasses
:   OntClass::listSubClasses() in OntClass.php

    Answer an array over the classes that are declared to be sub-classes of this class.

listSubProperties
:   OntProperty::listSubProperties() in OntProperty.php

    Answer an array of all the properties that are declared to be sub-properties of this property. Each element of the iterator will be an OntProperty.

listSuperClasses
:   OntClass::listSuperClasses() in OntClass.php

    Answer an array over the classes that are declared to be super-classes of this class.

listSuperProperties
:   OntProperty::listSuperProperties() in OntProperty.php

    Answer an array of all the properties that are declared to be super-properties of this property. Each element of the iterator will be an OntProperty.

m

top

MAX\_CARDINALITY
:   OWLVocabulary::MAX\_CARDINALITY() in OWLVocabulary.php

MIN\_CARDINALITY
:   OWLVocabulary::MIN\_CARDINALITY() in OWLVocabulary.php

n

top

NAMESPACE
:   OWLVocabulary::NAMESPACE() in OWLVocabulary.php

NAMESPACE
:   RdfsVocabulary::NAMESPACE() in RdfsVocabulary.php

    Answer the string that is the namespace prefix for this vocabulary

NOTHING
:   OWLVocabulary::NOTHING() in OWLVocabulary.php

o

top

OBJECT\_CLASS
:   OWLVocabulary::OBJECT\_CLASS() in OWLVocabulary.php

OBJECT\_PROPERTY
:   OWLVocabulary::OBJECT\_PROPERTY() in OWLVocabulary.php

OBJECT\_RESTRICTION
:   OWLVocabulary::OBJECT\_RESTRICTION() in OWLVocabulary.php

ONE\_OF
:   OWLVocabulary::ONE\_OF() in OWLVocabulary.php

ONTCLASS
:   RdfsVocabulary::ONTCLASS() in RdfsVocabulary.php

    Answer the resource that represents the class 'class' in this vocabulary.

OntClass
:   OntClass in OntClass.php

    Class that represents an ontology node characterising a class description.

ONTCLASS
:   OWLVocabulary::ONTCLASS() in OWLVocabulary.php

    Answer the resource that represents the class 'class' in this vocabulary.

OntClass
:   OntClass::OntClass() in OntClass.php

    Constructor You can supply a uri

OntModel
:   OntModel::OntModel() in OntModel.php

    Constructor.

OntModel
:   OntModel in OntModel.php

    Enhanced view of the model that is known to contain ontology data, under a

OntClass.php
:   OntClass.php in OntClass.php

OntModelP.php
:   OntModelP.php in OntModelP.php

OntModel.php
:   OntModel.php in OntModel.php

OntProperty.php
:   OntProperty.php in OntProperty.php

OntResource.php
:   OntResource.php in OntResource.php

OntVocabulary.php
:   OntVocabulary.php in OntVocabulary.php

OWLVocabulary.php
:   OWLVocabulary.php in OWLVocabulary.php

ONTOLOGY
:   OWLVocabulary::ONTOLOGY() in OWLVocabulary.php

OntProperty
:   OntProperty::OntProperty() in OntProperty.php

    Constructor.

OntProperty
:   OntProperty in OntProperty.php

    Class encapsulating a property in an ontology.

OntResource
:   OntResource::OntResource() in OntResource.php

    Constructor You can supply a uri

OntResource
:   OntResource in OntResource.php

    Provides a common super-type for all of the abstractions in this ontology representation package.

OntVocabulary
:   OntVocabulary in OntVocabulary.php

    Base Class that encapsulates the elements of a general vocabulary

ON\_PROPERTY
:   OWLVocabulary::ON\_PROPERTY() in OWLVocabulary.php

OWLVocabulary
:   OWLVocabulary in OWLVocabulary.php

    OWL vocabulary items

p

top

PRIOR\_VERSION
:   OWLVocabulary::PRIOR\_VERSION() in OWLVocabulary.php

PROPERTY
:   OWLVocabulary::PROPERTY() in OWLVocabulary.php

r

top

RdfsVocabulary.php
:   RdfsVocabulary.php in RdfsVocabulary.php

RANGE
:   OWLVocabulary::RANGE() in OWLVocabulary.php

    Answer the predicate that denotes the domain of a property.

RANGE
:   RdfsVocabulary::RANGE() in RdfsVocabulary.php

    Answer the predicate that denotes the domain of a property.

RdfsVocabulary
:   RdfsVocabulary in RdfsVocabulary.php

    RDFS vocabulary items

removeComment
:   OntResource::removeComment() in OntResource.php

    Remove the statement that the given ResLiteral is a comment on this resource.

removeDefinedBy
:   OntResource::removeDefinedBy() in OntResource.php

    Remove the statement that this resource is defined by the given resource.

removeDomain
:   OntProperty::removeDomain() in OntProperty.php

    Remove the given class from the stated domain(s) of this property.

removeLabelProperty
:   OntResource::removeLabelProperty() in OntResource.php

    Remove the statement that the given ResLiteral is a label on this resource.

removeProperty
:   OntResource::removeProperty() in OntResource.php

    Remove the specific property-value pair from this resource.

removeRange
:   OntProperty::removeRange() in OntProperty.php

    Remove the given class from the stated range(es) of this property.

removeRDFType
:   OntResource::removeRDFType() in OntResource.php

    Remove the statement that this resource is of the given RDF type.

removeSeeAlso
:   OntResource::removeSeeAlso() in OntResource.php

    Remove the statement indicating the given resource as a source of additional information about this resource.

removeSubClass
:   OntClass::removeSubClass() in OntClass.php

    Remove the given class from the sub-classes of this class.

removeSubProperty
:   OntProperty::removeSubProperty() in OntProperty.php

    Remove the given property from the sub-properties of this property.

removeSuperClass
:   OntClass::removeSuperClass() in OntClass.php

    Remove the given class from the super-classes of this class.

removeSuperProperty
:   OntProperty::removeSuperProperty() in OntProperty.php

    Remove the given property from the super-properties of this property.

RESTRICTION
:   OWLVocabulary::RESTRICTION() in OWLVocabulary.php

s

top

SAME\_AS
:   OWLVocabulary::SAME\_AS() in OWLVocabulary.php

SAME\_CLASS\_AS
:   OWLVocabulary::SAME\_CLASS\_AS() in OWLVocabulary.php

SAME\_INDIVIDUAL\_AS
:   OWLVocabulary::SAME\_INDIVIDUAL\_AS() in OWLVocabulary.php

SAME\_PROPERTY\_AS
:   OWLVocabulary::SAME\_PROPERTY\_AS() in OWLVocabulary.php

SEE\_ALSO
:   OWLVocabulary::SEE\_ALSO() in OWLVocabulary.php

    Answer the predicate that denotes seeAlso annotation on an ontology element

SEE\_ALSO
:   RdfsVocabulary::SEE\_ALSO() in RdfsVocabulary.php

    Answer the predicate that denotes seeAlso annotation on an ontology element

setComment
:   OntResource::setComment() in OntResource.php

    Assert that the given string is the comment on this resource.

setDomain
:   OntProperty::setDomain() in OntProperty.php

    Assert that the given resource represents the class of individuals that form the domain of this property. Any existing domain statements for this property are removed.

setInstanceRdfType
:   OntResource::setInstanceRdfType() in OntResource.php

    Sets the rdf:type, that this distinct resource is instance of.

setIsDefinedBy
:   OntResource::setIsDefinedBy() in OntResource.php

    Assert that the given resource provides a source of definitions about this resource.

setLabelProperty
:   OntResource::setLabelProperty() in OntResource.php

    Assert that the given string is the label on this resource.

setPropertyValue
:   OntResource::setPropertyValue() in OntResource.php

    Set the value of the given property of this ontology resource to the given value.

setRange
:   OntProperty::setRange() in OntProperty.php

    Assert that the given resource represents the class of individuals that form the range of this property. Any existing range statements for this property are removed.

setRDFType
:   OntResource::setRDFType() in OntResource.php

    Set the RDF type (ie the class) for this resource, replacing any existing rdf:type property. Any existing statements for the RDF type will first be removed.

setSeeAlso
:   OntResource::setSeeAlso() in OntResource.php

    Add a resource that is declared to provided additional information about the definition of this resource

setSubClass
:   OntClass::setSubClass() in OntClass.php

    Assert that this class is super-class of the given class.

setSubProperty
:   OntProperty::setSubProperty() in OntProperty.php

    Assert that this property is super-property of the given property.

setSuperClass
:   OntClass::setSuperClass() in OntClass.php

    Assert that this class is sub-class of the given class.

setSuperProperty
:   OntProperty::setSuperProperty() in OntProperty.php

    Assert that this property is sub-property of the given property.

setVocabulary
:   OntResource::setVocabulary() in OntResource.php

    Sets the reference to the assoiated vocabulary

SOME\_VALUES\_FROM
:   OWLVocabulary::SOME\_VALUES\_FROM() in OWLVocabulary.php

SUB\_CLASS\_OF
:   RdfsVocabulary::SUB\_CLASS\_OF() in RdfsVocabulary.php

    Answer the predicate that denotes that one class is a sub-class of another.

SUB\_CLASS\_OF
:   OWLVocabulary::SUB\_CLASS\_OF() in OWLVocabulary.php

    Answer the predicate that denotes that one class is a sub-class of another.

SUB\_PROPERTY\_OF
:   RdfsVocabulary::SUB\_PROPERTY\_OF() in RdfsVocabulary.php

    Answer the predicate that denotes that one property is a sub-property of another.

SUB\_PROPERTY\_OF
:   OWLVocabulary::SUB\_PROPERTY\_OF() in OWLVocabulary.php

    Answer the predicate that denotes that one property is a sub-property of another.

SYMMETRIC\_PROPERTY
:   OWLVocabulary::SYMMETRIC\_PROPERTY() in OWLVocabulary.php

t

top

THING
:   OWLVocabulary::THING() in OWLVocabulary.php

TRANSITIVE\_PROPERTY
:   OWLVocabulary::TRANSITIVE\_PROPERTY() in OWLVocabulary.php

TYPE
:   RdfsVocabulary::TYPE() in RdfsVocabulary.php

    Answer the predicate that denotes the rdf:type property.

TYPE
:   OWLVocabulary::TYPE() in OWLVocabulary.php

    Answer the predicate that denotes the rdf:type property.

u

top

UNION\_OF
:   OWLVocabulary::UNION\_OF() in OWLVocabulary.php

v

top

VERSION\_INFO
:   OWLVocabulary::VERSION\_INFO() in OWLVocabulary.php

a
b
c
d
e
f
g
h
i
l
m
n
o
p
r
s
t
u
v
